# Supplementary material for: Efficacy analysis of new copper complex for visible light (455, 530 nm) radical/cationic photopolymerization: The synergic effects and catalytic cycle
Source: PLoS One. 2022 Jul 26;17(7):e0270679. doi: 10.1371/journal.pone.0270679 (PMC9321381; doi:10.1371/journal.pone.0270679)
Supplement: Attachment — Submitted filename: REPLY to cmmments.docx [file pone.0270679.s001.docx]

[Additional Editor Comments], noting that there are NO comments from the Reviewers.

As it stands, this paper does not meet the criteria for original research. The manuscript presents the derivation of several mathematical models that describe the kinetics of an A/B/N type free radical polymerisation, based on a mechanism proposed elsewhere. The model is relevant and useful to prediction of reaction outcomes.

(1) However, there are no results (either experimental or theoretical) to explore the validity of the model in this paper. There is also no discussion as to whether the kinetics this model predicts make physical sense or show the proposed mechanism to be accurate. The conclusions drawn under the analytical results section (that the co-initiators B and N have multiple functions, that the CE has a transient state proportional to t, etc) are not conclusions that are proven by the model. Rather the mathematical model assumes the mechanism that these conclusions are based on. For these reasons the paper is closer to a 'hypothesis' paper as it stands.

(2) In addition the manuscript very similar in content to another article in the system (PONE-D-21-40218). I believe these articles could readily be combined into a single manuscript in accordance with PLOS one's policy on similar submissions. This would make a very interesting theoretical paper, if the model outputs under various reagent concentrations used to show the validity (or at least provide testable predictions) of the proposed mechanism.

our REPLY:

(1) We disagreed with Editor's comments, which might have mis-read sections of our manuscript. In fact,the measured data of Mokbel et al [16] and *Mau* et al [17] are quantitatively analyzed by our formulas, referred to sections 3.2 and 3.4 ***Analysis of measured data (G1 and G2 systems)***

(2) We totally agreed with Editor suggestion, and have combined 2 articles in one.
