# Supplementary material for: Efficacy analysis of new copper complex for visible light (455, 530 nm) radical/cationic photopolymerization: The synergic effects and catalytic cycle
Source: PLoS One. 2022 Jul 26;17(7):e0270679. doi: 10.1371/journal.pone.0270679 (PMC9321381; doi:10.1371/journal.pone.0270679)
Supplement: Attachment — Submitted filename: REPLY to Comments.docx [file pone.0270679.s002.docx]

Reply to academic editor comments:

1. we fixed style to meet PLosone.

2. we added DOI for refs.

3. in Figs 1 and 2, (and in the text) we cited Ref. [18] for overlaping text. In fact,

this article was written before Ref. [18] (a review article) was published. we cited Ref [18] in many places.

4. see revised cover letter for Funding inform. pls do online submission form on our behalf.

5. we move the funding inform from Acknowledgement.

6. see 5.

Additional comments:

a) the readability needs to be improved to better explain how the equations have been derived and;

Our Reply: more text was added (shown in red).

b) the correlation between these equations and published data needs to be better shown to demonstrate the validity of these results.

Our Reply: more text was added (shown in red).

Rev.#1,

- - section 2.1 and 2.2 should be clarify for a better understanding of the developed formulas
  - more correlation between the developed formulas and the published experimental data would be a great addition in section 3.1 and 3.2 in order to prove the trustworthiness of the formulas and their usefulness for simulation.
- REPLY: detailed comparison of experimental curves and our modeling require a complex numerical solutions, which is out of the scope of the present article (which focuses on simplified but analytic formulas).
- Moreover, many of the rate constants (kj, Kj) in our modeling are not yet available (measured), therefore, it is impossible to have pertinent figures for a direct comparing to the measured curved. If the related rate constants are given (measured) then our derived formulas will lead to numerical roles of the key parameters, which are quantitative (not qualitative). Our developed simplified formulas, however, provide important features explored experimentally.
- -L99, 156, 172 & 183: The authors refer to the active species for cationic polymerization (either "S" or "EDB(+)") as a radical, are you sure that it is not a cation radical?
- REPLY: yes, agreed and fixed. Many thanks.
- -L205: The reaction r6 in scheme 4 seems unbalanced
- REPLY: yes, we fixd it by EDB^o^(+)
- -L218: What phenomenon is represented by the rate constant K12? Please add more explanation.

REPLY: we add these text: In Eq. (17) and (18), K12 is the coupling between Iod (B) and EDB (N), which also produces extra radical R (without the light), as shown by our r6. However the FRP due to this extra R is very small (for a week coupling constant of K12), as reported in Ref [17].

-L251: The authors claim the conversion of the cationic polymerization is reduced due to oxygen inhibition. However, cationic polymerization is reported in the literature, as not sensitive to oxygen. Please, justify your claim.

REPLY: agreed and fixed.

L272: The authors refer to the concentration of [A] in Eq 16, please clarify.

REPLY: corrected as Eq. (19).

-L374: (386) Where does this formula come from. Please explain or add a reference. REPLY: we added Ref. [21,22].

- -L505: Why have the authors consider the case of Iod/EDB without light?Please explain.

REPLY: the Iod/EDB was used as a standard to ensure that the EDB/Iod charge transfer complex could not initiate the polymerization, if lack of absorption for the charge transfer complex at a non-absorbing light wavelength (or no light). It was also measured in ref [17].

- -L627: The authors have linked the difference in conversion between the free radical polymerization and the cationic polymerization to the number of species capable to initiate the reactions (2 for FRP, 1 for CP). However, the monomers used in the experimental data, TMPTA and EPOX, does not have the same functionality (TMPTA is a trifunctional monomer while EPOX is a difunctional monomer). Moreover, FRP and CP usually have not the same rate of polymerization. Please, include these facts in your explanation.
- REPLY: yes, we added text in L 676-679.

Rev.#2 ***********************************************************

- 1) For both G1 and G2, while the claim is made for a quantitative agreement with experiment, in fact only qualitative features are compared, increases or decreases etc. Reference is made to various figures from Refs. 16 and 17 and the reader is expected to follow the described trends mentally, imagining the figures. I would strongly suggest that pertinent figures be adapted to show both the experimental and the authors' approximate analytical solutions so that the reader can actually compare them.
- REPLY: a detailed comparison of experimental curves and our modeling require a complex numerical solutions, which is out of the scope of the present article (which focuses on simplified but analytic formulas).
- Moreover, many of the rate constants (kj, Kj) in our modeling are not yet available (measured), therefore, it is impossible to have pertinent figures for a direct comparing to the measured curved. See more discussion in our REPLY after comments 9).
- 2) The manuscript is the result of the fusion of two previous manuscripts and many errors have been made in the process. These must be carefully checked and corrected.
- Yes, we have switched the definition of G1 and G2, such that G1 is for the first-system and G2 is for the second system to avoid errors caused by them.
- Just a few examples
- (1) (line 169, Scheme 3 should be Scheme 4; In Scheme 3, it should be G2, both in the scheme and in the legend;
- REPLY: fixed as G1, we have switched G2 and G1.
- (2) line 184, (r6) of Scheme 2 should be (r6) of Scheme 4; fixed
- (3) line 194, Scheme 1 should be Scheme 2); fixed
- (4) line 272, constant [A] in Eq. (16) should refer to Eq. (20); fixed
- (5) line 300 (12) and (13) are for G2 but this section is on G1, etc, etc).
- REPLY: fixed. we have switched G1 and G2 for consistent.
- 6) Throughout the manuscript, replace oxodized by oxidized.
  7) line 242, line 274, I don't think these are "comprehensive formulas". They are simplified formulas for some limiting cases. partially agreed and fixed
  8) line 291, S and S should be S and S' fixed
  9) lines 294 to 400, just one example of qualitative feature being misconstrued as quantitative: " faster raising rate".
- REPLY: we partially agreed. In fact, the discussions shown in sections 3.2 (for G1 system) and 3.4 (for G2 system) are based on our analytic formulas which defined specifically and quantitatively the roles of each of the key parameters for the conversion efficacy. Our formulas predict many of the measured data (and their general trends), besides other to-be-explored features. If the related rate constants such as kj and Kij, are given (measured) then our derived formulas will lead to numerical roles of the key parameters, which are quantitative (not qualitative).
