# Supplementary material for: Efficacy analysis of new copper complex for visible light (455, 530 nm) radical/cationic photopolymerization: The synergic effects and catalytic cycle
Source: PLoS One. 2022 Jul 26;17(7):e0270679. doi: 10.1371/journal.pone.0270679 (PMC9321381; doi:10.1371/journal.pone.0270679)
Supplement: Attachment — Submitted filename: comment-plos one.docx [file pone.0270679.s003.docx]

Journal Requirements:

Additional Editor Comments (if provided):

The authors have responded to the first point (a) raised by the reviewers - the revised manuscript is more readable and the derivations better eplained. The derivations are detailed and accurate (as they were in the original submission). However, they have still not answered the main comment raised by both revieiwers - that there is no comparison of the results of the analytical solutions with experimental data.
Producing such figures (even in the absence of experimental data, to show the general trends align, or perhaps on just one set of input initiator values) should be a matter of hours and would completely put to rest this question? If this is not the case, can the authors please explain why not??

REPLY: yes, we agreed and we have added Section 3.5 to include figs 1 to 3 to show the roles of key parameters, which are related to figs (measured data) of prior articles.

Minor typos to fix: (all fixed in "red")
L133: “coupling” not “conpling”
L135: “example” not “examples"
L446: “similar to THE G1 system of 3.1”
L668: “Eqs.(“ missing text
